# Molecular Features of Glioma Determined and Validated Using Combined TCGA and GTEx Data Analyses

**DOI:** 10.3389/fonc.2021.729137

**Published:** 2021-09-30

**Authors:** Zijiang Yang, Weiyi Gong, Ting Zhang, Heng Gao

**Affiliations:** ^1^ Department of Neurosurgery, Affiliated Kunshan Hospital of Jiangsu University, Kunshan, China; ^2^ Department of Central Laboratory, Jiangyin Clinical College of Xuzhou Medical College, Jiangyin, China; ^3^ Department of Neurosurgery, Jiangyin Clinical College of Xuzhou Medical College, Jiangyin, China

**Keywords:** glioma, prognostic biomarker, gene expression, immune cell, gene-targeted drug

## Abstract

Gliomas are among the most common intracranial tumors which originated from neuroepithelial cells. Increasing evidence has revealed that long noncoding RNA (lncRNA)-microRNA (miRNA)-mRNA module regulation and tumor-infiltrating immune cells play important regulatory roles in the occurrence and progression of gliomas. However, the precise underlying molecular mechanisms remain largely unknown. Data on gliomas in The Cancer Genome Atlas lack normal control samples; to overcome this limitation, we combined 665 The Cancer Genome Atlas glioma RNA sequence datasets with 188 Genotype-Tissue Expression normal brain RNA sequences to construct an expression matrix profile after normalization. We systematically analyzed the expression of mRNAs, lncRNAs, and miRNAs between gliomas and normal brain tissues. Kaplan–Meier survival analyses were conducted to screen differentially expressed mRNAs, lncRNAs, and miRNAs. A prognostic miRNA-related competitive endogenous RNA network was constructed, and the core subnetworks were filtered using 6 miRNAs, 3 lncRNAs, and 11 mRNAs. Gene Ontology and Kyoto Encyclopedia of Genes and Genomes enrichment analyses were performed to investigate the biological functions of significantly dysregulated mRNAs. Co-expression network analysis was performed to analyze and screen the core genes. Furthermore, single-sample Gene Set Enrichment Analysis and immune checkpoint gene expression analysis were performed, as co-expression analysis indicated immune gene dysregulation in glioma. Finally, the expression of representative dysregulated genes was validated in U87 cells at the transcriptional level, establishing a foundation for further research. We identified 7017 mRNAs, 437 lncRNAs, and 9 miRNAs that were differentially expressed in gliomas. Kaplan–Meier survival analysis revealed 5684 mRNAs, 61 lncRNAs, and 7 miRNAs with potential as prognostic signatures in patients with glioma. The hub subnetwork of the competing endogenous RNA network between PART1-hsa-mir-25-SLC12A5/TACC2/BSN/TLN2/ZDHHC8 was screened out. Gene co-expression network, single-sample Gene Set Enrichment Analysis, and immune checkpoint expression analysis demonstrated that tumor-infiltrating immune cells are closely related to gliomas. We identified novel potential biomarkers to predict survival and therapeutic targets for patients with gliomas based on a large-scale sample. Importantly, we filtered pivotal genes that provide valuable information for further exploration of the molecular mechanisms underlying glioma tumorigenesis and progression.

## Introduction

Glioma, classically classified in four WHO grades, is the most common malignant neoplasm among primary central system tumors with high mortality (1). A higher glioma grade is associated with a poorer patient prognosis. Recent years, based on molecular markers, such as IDH mutation, 1p/19q codeletion, Histone H3 K27M mutation, Histone H3.3 G34R/V mutation, MGMT et.al, diffuse gliomas were divided into oligodendroglioma IDH-mutation and 1p/19q codeletion, WHO grade II-III; Astrocytoma, IDH-mutation, WHO grade II-III; Astrocytoma, IDH-mutation, WHO grade IV; Glioblastomas, IDH wild type, MGMT promoter methylation, WHO grade IV; Diffuse hemispheric gliomas, H3.3 G34-mutant, MGMT promoter methylation, WHO grade IV; Diffuse midline gliomas, H3 K27M-mutant, WHO grade IV (2). Different diffuse glioma types lead to different prognosis and treatment methods. Researchers showed that greater grading was associated with poorer prognosis and was an independent prognostic factor in IDH-mutant grade II and grade III gliomas (3). Gliomas grading and molecular characteristics all should be considered into patients’ management for improving prognosis (4). Glioblastoma (GBM), which is a World Health Organization grade IV tumor account for 53.1% of all intracranial neuroepithelial tumors with a median survival of 8 months and 5-year survival rate of 7.2% (1), is newly defined as a diffuse astrocytoma without IDH nor histone H3 genes mutation. It is characterized by microvascular hyperplasia, necrosis and/or specific molecular characteristics, including TERT promoter mutations, EGFR gene amplification, and/or a + 7/- 10 cytogenetic feature (2). Despite continuous progress in glioma diagnostic and therapeutic strategies, the mortality rate of glioma remains high. This high morbidity is associated with high tumor heterogeneity, early and widespread diffuse malignant cell infiltration, difficulty in achieving complete surgical removal, and high intrinsic chemotherapy and radiotherapy resistance (5). However, the molecular mechanisms underlying gliomas remain unclear. Therefore, studies with regard to the molecular mechanism of gliomas are urgently needed to identify potential prognostic molecular markers and effective therapeutic targets to improve the survival of patients with glioma.

Long noncoding RNAs (lncRNAs) are the largest class of noncoding RNAs; these sequences are composed of more than 200 nucleotides and have rich biological functions. LncRNAs play crucial regulatory roles in modulating the cell cycle, cell proliferation, cell invasion, and migration by regulating gene expression at the transcriptional, post-transcriptional, and epigenetic levels (6, 7). Studies have demonstrated that dysregulation of lncRNA expression is related to gliomas tumorigenesis and cancer progression and that it is a potentially effective early diagnostic, prognostic biomarker, and therapeutic target (8). However, the specific and detailed molecular mechanisms underlying the involvement of lncRNAs in glioma carcinogenesis and progression remain largely unknown.

MicroRNAs (miRNAs) are endogenous noncoding RNAs with a length of 20–22 nucleotides and regulate gene and transcription factor expression at the transcriptional and post-transcriptional levels. MiRNAs are closely related to the tumor microenvironment, which comprises various cell types and molecular patterns and, in turn, affects cancer cells and cancer-related miRNA expression. Many studies have shown that miRNAs also play important roles in tumorigenic processes, such as cell proliferation, metastasis, invasion, and angiogenesis (9).

LncRNAs act as microRNA sponges *via* miRNA-response elements and reverse the inhibition of miRNAs and target mRNAs to function as competing endogenous RNAs (ceRNAs) (10). An increasing number of reports have shown that the ceRNA module is involved in tumor initiation and progression. However, limited ceRNA research has been conducted on gliomas.

This study was conducted to explore the molecular mechanisms underlying the coding and noncoding RNAs of gliomas. We comprehensively analyzed the association of mRNA, lncRNA, and miRNA expression profiles and clinical outcomes in a large cohort of patients with glioma by combining The Cancer Genome Atlas (TCGA) RNA-seq data with Genotype-Tissue Expression (GTEx) normal brain RNA-seq data, as TCGA glioma data lack normal control samples. We first constructed a lncRNA-miRNA-mRNA ceRNA network with prognostic value for patients with glioma. Significant differential expression of representative lncRNAs, miRNAs, and mRNAs was further confirmed at the cellular level. To further explore the molecular mechanisms of a glioma, the functions of differentially expressed genes (DEGs) were evaluated. Gene co-expression and transcription factor (TF) prediction was conducted to clarify the regulatory relationships between DEGs.

Currently, the tumor immune environment and immune-related treatments have received increased attention. Immune cell and pathway deregulation has been studied in glioma using single-sample Gene Set Enrichment Analysis (ssGSEA). We analyzed the expression of immune checkpoint (IC) molecules (IDO, TIM-3, LAG-3, CTLA-4 (CD152), PD-1, PD-L1, PD-L2, B7-H3, B7-H4, B7-H5, and TIGIT) to provide a theoretical basis for tumor immunotherapy. Furthermore, we filtered small molecular drug targets on DEGs to identify potential treatments using the Drug Gene Interaction Database (DGIDB). These emerging strategies indicate the potential of tumor immunotherapy to overcome the limitations of existing tumor immunotherapy. It is hoped that our data will reveal the mechanisms underlying the regulation of glioma development and prognosis and provide novel prognostic biomarkers and therapeutic targets for glioma.

## Materials and Methods

### TCGA and GTEx Raw Data

RNA sequencing and corresponding clinical data associated with gliomas were retrieved from TCGA database (Data Release 27.0). Normal brain RNA sequencing data were downloaded from GTEx and used as normal control data. Glioma patients’ corresponding clinical data were summarized in **Supplementary Table 1**. We collected 665 glioma samples and 188 normal brain samples to investigate significant changes in lncRNAs, miRNAs, mRNAs, and their complex interactions in relation to carcinogenesis after integration and normalization using the limma package (11).

### Screening of Differentially Expressed (DE) mRNAs, lncRNAs, and miRNAs and ceRNA Network Construction

The limma package was used to filter the DE mRNAs, lncRNAs, and miRNAs between the glioma and normal brain tissues with the criterion of |log2 fold-change| ≥2.0 and false discovery rate p-value of <0.05 (11). Survival curve analysis of DE lncRNAs, miRNAs, and mRNAs was performed using the survival package. The expression of miRNAs, mRNAs, and lncRNAs was presented in a heatmap. Prognosis-related DEmiRNAs were screened out to construct the ceRNA network based on the hypothesis that lncRNAs directly interact with sequence complementary miRNAs that regulate targeted mRNA expression. Thus, lncRNA–prognostic miRNA and prognostic miRNA-mRNA interactions were predicted. LncRNA–prognostic miRNA interactions were predicted using the miRcode database, whereas prognostic miRNA-mRNA interactions were predicted using the miRDB, miRTarBase, and TargetScan databases. mRNAs of prognostic miRNA targets intersecting with DEmRNAs were used in the next ceRNA network. After obtaining the lncRNA–prognostic miRNA-mRNA interactions, Cytoscape3.6.0 was used to visualize the lncRNA–miRNA–mRNA ceRNA network.

### DEmRNA Co-Expression and DEmRNA TF Prediction

Gene co-expression network analysis reveals interactions between gene molecules and can reflect the regulation of gene expression at a precise level. The DEmRNA expression matrix file was used to calculate the corweight, and the co-expression network was visualized using the igraph package and Cytoscape. TFs were predicted and visualized using chea3 online (https://maayanlab.cloud/chea3/#top).

### Gene Ontology (GO) and Kyoto Encyclopedia of Genes and Genomes (KEGG) Functional Enrichment Analyses

GO and KEGG analyses were performed to predict the functions of DEmRNAs using the clusterProfiler package (12). Fisher’s test was used to identify significant (p < 0.05) and significantly enriched function annotations (p < 0.05).

### Glioma Immune and Related Small Molecular Drug Prediction

The ssGSEA algorithm was used to evaluate the degree of immune infiltration in the glioma microenvironment. mRNA expression data were compared with the gene set using “GSVA” in the R package. The following 11 types of immune cells were obtained: activated dendritic cells, B cells, CD8+T cells, macrophages, neutrophils, plasmacytoid dendritic cells, Th1 cells, Th2 cells, T helper cells, tumor-infiltrating lymphocytes, and regulatory T cells (Tregs). Furthermore, the following 13 immune functions were obtained: APC_co_inhibition, APC_co_stimulation, CCR, checkpoint, cytolytic activity, human leukocyte antigen (HLA), inflammation-promoting, MHC_class_I, parainflammation, T_cell_co-inhibition, T_cell_co-stimulation, Type_I_IFN_Reponse, and Type_II_IFN_Response. * represented p < 0.05, *** represented p < 0.01. The expression of ICs (B7-H3, B7-H4, B7-H5, IDO, LAG-3, PD-1, PD-L1, PD-L2, and TIM-3) was analyzed using the limma package. A p value of <0.05 was considered to indicate statistically significant results. The top 100 DEmRNAs were selected to identify target drugs in DGIDB (https://dgidb.genome.wustl.edu/) and visualized using Cytoscape.

### Quantitative Reverse Transcription Polymerase Chain Reaction (qRT-PCR) Validation of Representative Genes in U87 Cells

U87 cells and normal brain tissues were used for qRT-PCR. Total RNA was isolated from cells and brain tissues. RR036A reverse transcriptase (Takara, Shiga, Japan) was used to synthesize cDNA. qRT-PCR was performed using a Bio-Rad Cfx96 system (Hercules, CA, USA). Glyceraldehyde 3-phosphate dehydrogenase was used as an internal reference to quantify the mRNAs. qRT-PCR assays were performed in triplicate. Primer and probe sequences of the genes used for qRT-PCR analysis are listed in **Supplementary Table 2**.

## Results

### Identification of DEmRNAs, DElncRNAs, and DEmiRNAs With Prognostic Significance

**Figure 1:**
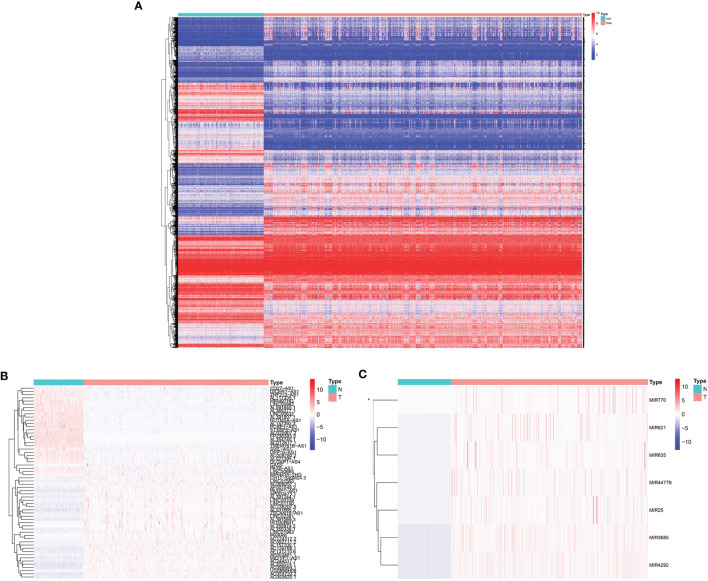
Heatmap with prognostic differentially expressed genes (DEGs). **(A)** DE prognostic mRNAs. **(B)** DE prognostic long noncoding RNAs (lncRNAs). **(C)** DE prognostic microRNAs.

In total, 665 glioma samples from TCGA RNA sequencing data were compared with 188 normal brain RNA sequencing datasets from GTEx after normalization. Data processing identified 7017 mRNAs, 437 lncRNAs, and 9 miRNAs that were significantly DE (|log2 fold-change| ≥2.0 and false discovery rate p < 0.05, shown in **Supplementary Table 3**). Survival analysis was conducted with the DE genes combined with the corresponding clinical data using Kaplan-Meier curves; 5684 mRNAs, 61 lncRNAs, and 7 miRNAs showed significantly altered expression with prognostic significance (**Supplementary Table 4**). The heatmap of clustering analysis of DE prognostic genes is shown in **Figures 1A**–**C**.

### Interactions Between lncRNAs, Prognostic miRNAs, and mRNAs

In total, 159 of 436 prognostic lncRNAs were matched to the miRcode database, and 138 pairs of lncRNA-prognostic miRNA interactions were predicted. Additionally, 2790 pairs of miRNA-mRNA interactions were predicted using the miRDB, miRTarBase, and TargetScan databases, and 1140 gene pairs remained after selecting microRNA target genes that intersected with the 5685 mRNAs.

### LncRNA-miRNA-mRNA ceRNA Network and Key Sub-Network Construction

**Figure 2:**
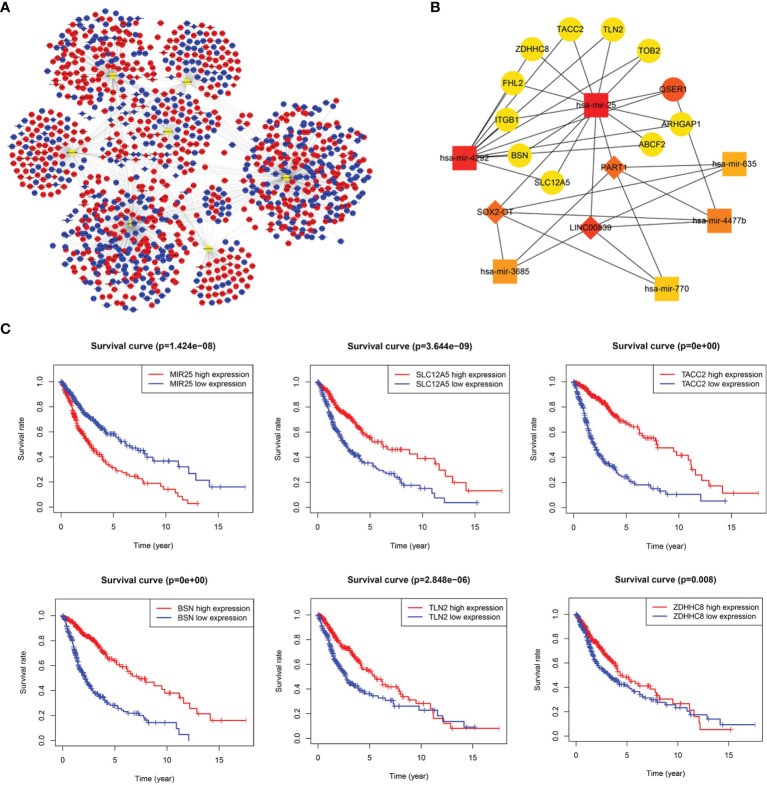
Long noncoding RNA (lncRNA)-prognostic microRNA (miRNA)-mRNA competing endogenous RNA (ceRNA) network in glioma. **(A)** Overview of the ceRNA network in glioma. Red and blue diamonds represent upregulated and downregulated lncRNAs, respectively. Red and blue circles represent upregulated and downregulated mRNAs, respectively. Orange rounded rectangles represent microRNAs. **(B)** Key sub-network of the ceRNA network. **(C)** Survival curves for miR-25 and its targets (SLC12A5, TACC2, BSN, TLN2, and ZDHHC8).

The ceRNA network was established based on lncRNA-miRNA and miRNA-mRNA interactions (**Supplementary Table 5**). The ceRNA network consisted of 74 lncRNA nodes, 7 miRNA nodes, and 1035 mRNA nodes, with a total of 1278 interactions (**Figure 2A**). The key sub-network was filtered with the top 20 nodes, as shown in **Figure 2B**, with 6 microRNAs, 3 lncRNAs, and 11 mRNAs. hsa-mir-25 was highly expressed in gliomas showing the highest rank, suggesting that it strongly contributes to glioma pathogenesis. LncRNA PART1 was downregulated and the target genes solute carrier family 12 member 5 (SLC12A5), transforming acidic coiled-coil 2 (TACC2), bassoon presynaptic cytomatrix protein (BSN), Talin 2 (TLN2), and zinc finger DHHC-type palmitoyltransferase 8 (ZDHHC8) all showed downregulated expression, which was associated with worse overall survival (OS). The Kaplan-Meier curve analysis is shown in **Figure 2C**. The regulation axis between PART1-hsa-mir-25-targets requires further exploration.

### DEG Co-Expression Network and DEG TF Prediction

**Figure 3:**
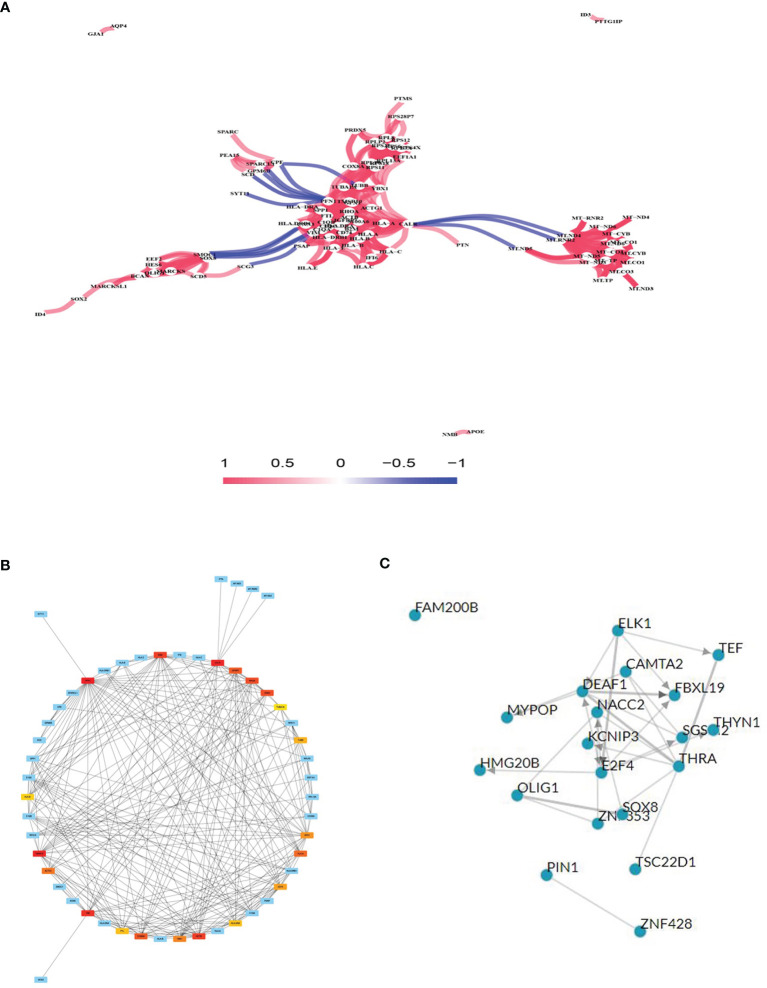
Gene co-expression network analysis and transcription factor (TF) prediction. **(A)** Gene co-expression network analysis with differentially expressed mRNAs. **(B)** Network of top 20 hub genes in the gene co-expression network. **(C)** TF prediction of differentially expressed mRNA.

Gene co-expression network analysis is a network graph constructed based on the similarity of gene expression data. Gene co-expression network analysis was conducted on the identified DEGs to determine possible gene interactions and identify core genes. The results showed that the HLA and mitochondrial transcription (MT) gene families were important in the network (**Figure 3A**). The top 20 co-expressed neighbors were selected, and the core genes PFN1, CALR, thymosin beta 10 (TMSB10), HLA.A, CD74 (HLADG), HLA.DRA, HLA.B, TUBB, and TUBA1A were found to play pivotal roles in the network, suggesting that immune genes are involved in glioma formation (**Figure 3B**). TFs are proteins that bind to DNA and regulate transcription by recognizing specific DNA sequences to form complex systems that direct gene expression. Chea3 was used to predict the TFs of DEGs, and potassium voltage-gated channel interacting protein 3 (KCNIP3), PIN1, and NACC2 were predicted to play crucial roles in regulating DEG expression (**Figure 3C**).

### mRNA GO and KEGG Pathway Analyses

**Figure 4:**
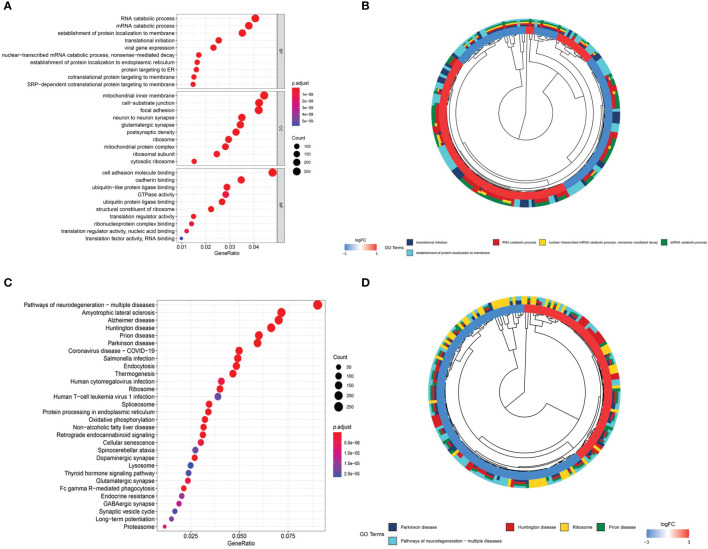
Gene Ontology (GO) and Kyoto Encyclopedia of Genes and Genomes (KEGG) enrichment analyses. **(A)** Bubble plot of GO enrichment analysis. **(B)** Circle plot of GO enrichment analysis. **(C)** Bubble plot of KEGG enrichment analysis. **(D)** Circle plot of KEGG enrichment analysis.

To better understand the role of DEGs in glioma involved in the ceRNA regulatory network, GO and KEGG pathway analyses were conducted. The top 10 GO and KEGG enrichment pathways are presented in **Figure 4**. The DEGs were mainly enriched in biological processes, including RNA catabolic process, mRNA catabolic process, establishment of protein localization to membrane, translational initiation, and viral gene expression, and cellular component analysis indicated enrichment in mitochondrial inner membrane, cell−substrate junction, focal adhesion, neuron to neuron synapse, and glutamatergic synapse. Molecular function enrichment included cell adhesion molecule binding, cadherin binding, ubiquitin-like protein ligase binding, GTPase activity, and ubiquitin protein ligase binding (**Figures 4A, B**). KEGG pathway enrichment analysis indicated the involvement of pathways influencing neurodegeneration, including multiple diseases, amyotrophic lateral sclerosis, Alzheimer’s disease, Huntington’s disease, prion disease, and Parkinson’s disease. All of these diseases are characterized by the presence of pathological changes in the central nervous system (**Figures 4C, D**).

### Glioma Immune and Related Small Molecular Drug Prediction

**Figure 5:**
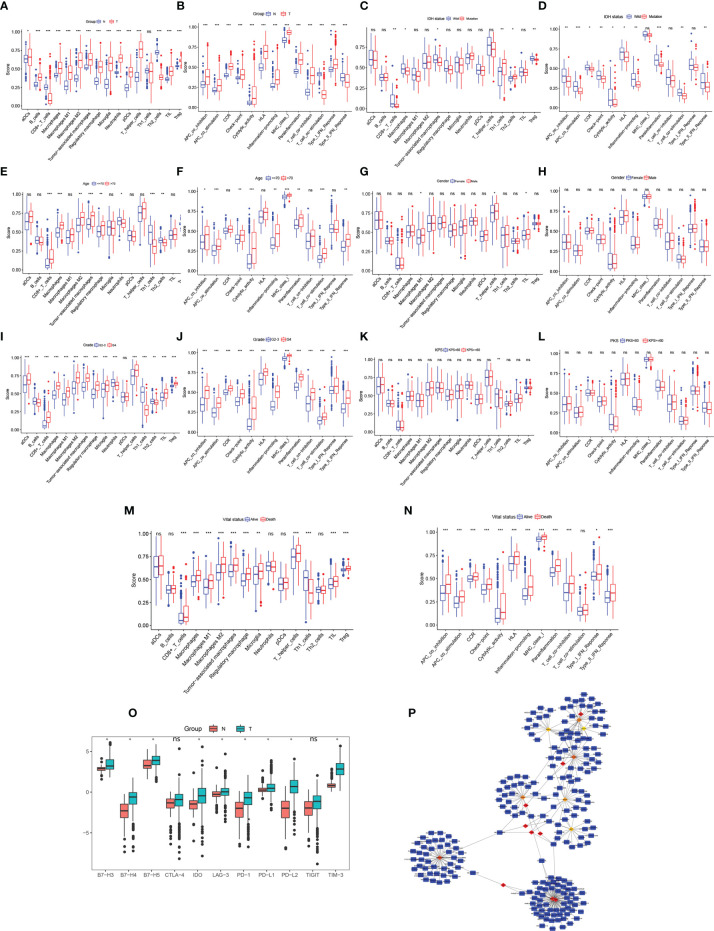
Immune cell exploration in gliomas and small molecular drug prediction. **(A, C, E, G, I, K, M)** Glioma immune cell analysis using single-sample Gene Set Enrichment Analysis (ssGSEA). **(B, D, F, H, J, L, N)** Glioma immune cell function analysis using ssGSEA. **(O)** Immune checkpoint expression in glioma compared to in the normal brain. **(P)** Small molecular drug prediction targeting pivotal genes. *p < 0.05, **p < 0.01, ***p < 0.001. ns, no statistical difference.

The results of the DEG co-expression network showed that the HLA family (HLA-A, HLA-B, HLA-C, HLA-E, HLA-DRA, HLA-DRB1, and CD74 [HLADG]) plays a vital role in the network, suggesting that tumor immunity is involved in glioma formation. The ssGSEA algorithm was used to evaluate the degree of immune infiltration between glioma and normal brain, IDH-wild and IDH-mutation, age<=70 and age>70, Male and Female, Grade2-3 and Grade4, KPS>=60 and KPS<60, Alive and Death subgroup. Immune cell results showed that the scores of activated dendritic cells, B cells, macrophages, macrophages M1, macrophages M2, tumor-associated macrophages, regulatory macrophage, microglia, neutrophils, plasmacytoid dendritic cells, T helper cells, and Tregs were significantly higher, whereas Th2 cells and CD8+T cells were significantly lower, in glioma samples than in normal brain samples (p < 0.05, **Figure 5A**). The scores for antigen-presenting cell co-stimulation, antigen-presenting cell co-inhibition, CCR, check point, cytolytic activity, HLA, inflammation-promoting, major histocompatibility complex class I, parainflammation, T cell co-inhibition, and type I interferon (IFN) response were higher, whereas those of T cell co-stimulation and type II IFN response were lower, in glioma samples than in normal brain samples (p < 0.05, **Figure 5B**). CD8+T cells, macrophages, tumor-associated macrophages and Treg were lower, whereas, Th1 cells and Th2 cells were significantly higher IDH-mutation samples (p < 0.05, **Figure 5C**). Antigen-presenting cell co-stimulation, antigen-presenting cell co-inhibition, CCR, check point, cytolytic activity, HLA, inflammation-promoting, parainflammation, T cell co-stimulation, and type II interferon (IFN) response were lower in IDH-mutation samples (p < 0.05, **Figure 5D**). In age greater than 70 years old group, CD8+_T_cells, macrophages, macrophages M2, tumor-associated macrophages, regulatory macrophage, showed higher infiltrated, neutrophils, Th1_cells, Th2_cells were lower infiltrated (p < 0.05, **Figure 5E**). Antigen-presenting cell co-stimulation, antigen-presenting cell co-inhibition, check-point, cytolytic activity, inflammation-promoting, major histocompatibility complex class I, parainflammation, T cell co-stimulation, type II IFN Reponse were activated in elder group (p < 0.05, **Figure 5F**). Dysregulation of immune cells and immune function were also found between G2-3 and G4 patients (**Figures 5I, J**), Alive and Death patients (**Figures 5M, N**). On the whole, KPS and gender of gliomas were not related to glioma immune (**Figures 5G, H, K, L**). ICs are a group of molecules expressed on immune cells and can regulate the degree of immune activation. ICs and their ligands are frequently upregulated in the tumor microenvironment of various malignancies and significantly block the induction of effective anti-tumor immune responses. The expression levels of B7-H3, B7-H4, B7-H5, IDO, LAG-3, PD-1, PD-L1, PD-L2, and TIM-3 were significantly higher in glioma samples than in normal brain samples (p < 0.05, **Figure 5O**), indicating their potential as therapeutic targets. Further, to identify potential therapeutic targets and drugs, the top 100 DEGs were selected and matched in DGIDB, and 17 hub genes with target drugs were screened out, including HLA family (HLA-A, HLA-B, and HLA-C) and TUB family (TUBB and TUBA1A) genes (**Figure 5P**). Ticlopidine, clavulanic acid, and amoxicillin, which target HLA-A, HLA-B, and HLA-C, and artenimol and colchicine, which target TUBB and TUBA1A, showed therapeutic potential.

### Validation of Representative Dysregulated DEGs in U87 Cells

**Figure 6:**
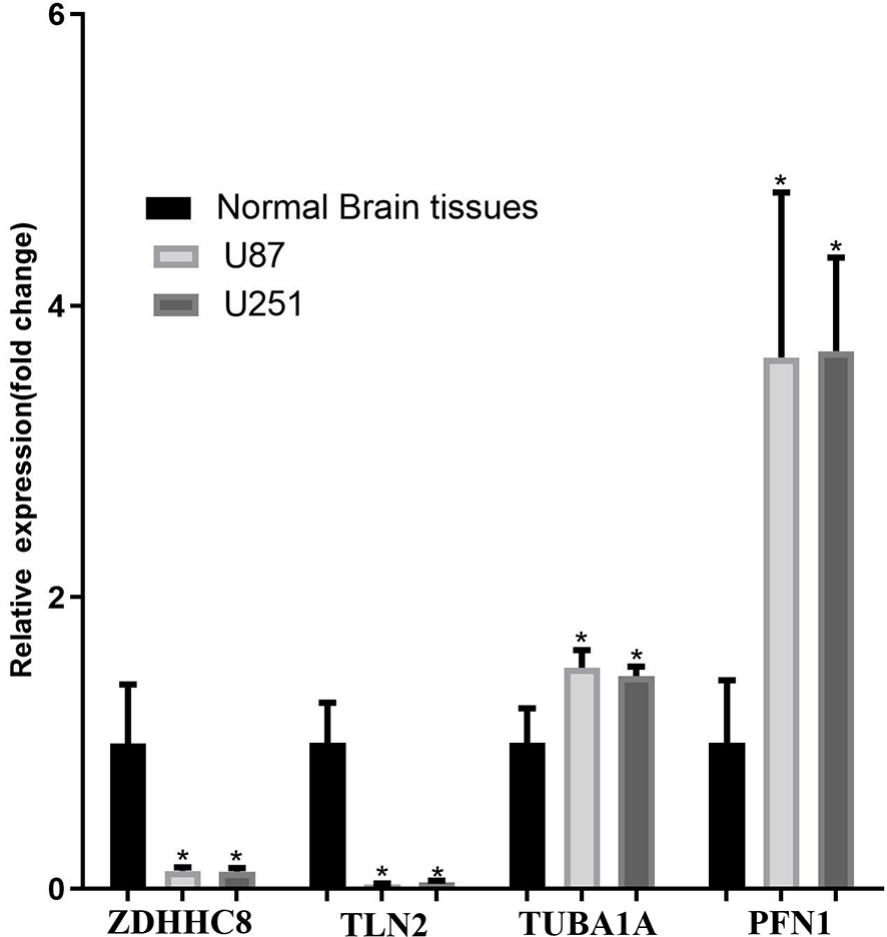
mRNA expression validation in U87 and U251 cells. ZDHHC8, TLN2, TUBA1A, PFN1 expression in U87 cells, U251 cells and normal brain. *p < 0.05.

The mRNA expression of ZDHHC8, TLN2 (hsa-mir-25 target gene), TUBA1A, PFN1 (the top 20 co-expressed genes) and PIN1 (TF of DEGs) was validated using U87 cells, U251and normal brain samples. The results revealed that ZDHHC8, TLN2 were significantly low expression and TUBA1A, PFN1, PIN1 were significantly higher expression in cancer (p < 0.05, **Figure 6**), which is consistent with our aforementioned results.

## Discussion

Glioma is a common malignant neuroepithelial neoplasm that contributes to the largest number of deaths caused by central nervous system tumors (13). WHO 2021 diffuse glioma classifications are relatively complex which based on histology and molecular markers, but it makes treatment more personalized (2). However, these precise treatments are after the accurate diagnosis. In order to improve the prognosis of glioma patients, early detection and treatment are urged needed. At present, the lack of early specific diagnostic and prognostic biomarkers and therapy resistance may contribute to the worse survival rate of patients with glioma compared with that of patients with other central nervous system tumors (5). Therefore, to improve clinical outcomes, it is essential to explore the exact intrinsic regulatory mechanisms of glioma initiation and progression and identify potential glioma-related prognostic signatures and new potential therapeutic targets. Research showed that circulating free DNA, circulating tumor cell, circulating tumor DNA and microRNA in blood or cerebrospinal fluid were used for GBM early diagnosis, early tumor progression detection, monitoring of tumor treatment response, personalized treatment identification, such as EGFR, TGF-β, IDH, miR-21and so on. Those gene play vital role in GBM early diagnosis and specific therapy and could be detected in liquid biopsy for overcoming the solid cancer biopsy limits (14).Recently, an increasing number of studies have demonstrated that miRNA-mediated ceRNA crosstalk plays a vital role in cancers, including glioma (9, 15).

As TCGA glioma data lack normal brain control data, comprehensive analyses of the DEmRNA and noncoding RNA profiles in glioma, based on large-scale RNA sequencing platforms, are lacking. Using RNA-seq data from TCGA and GTEx databases, we identified 7017 mRNAs, 437 lncRNAs, and 9 miRNAs that were aberrantly expressed in patients with glioma compared with normal brain tissues. Among these genes, 5684 mRNAs, 61 lncRNAs, and 7 miRNAs showed prognostic significance.

After constructing a ceRNA network and key sub-network, key genes were selected, and the results showed that the PART1-hsa-mir-25-SLC12A5/TACC2/BSN/TLN2/ZDHHC8 regulation axis plays an important role in the ceRNA network. LncRNA PART1 was downregulated in gliomas, and its overexpression in U87 cells induced cell growth arrest and apoptosis, indicating that it plays an anti-oncogenic role in glioma (16). Additionally, hsa-mir-25 was upregulated in gliomas and showed poor OS, which was consistent with the results of another study showing that miR-25 overexpression in U251 cells promotes cell proliferation and invasion (17). SLC12A5/TACC2/BSN/TLN2/ZDHHC8 was downregulated in patients with glioma with poor OS. SLC12A5 is a type of Kcl cotransporter that maintains neuronal chloride homeostasis, and its aberrant expression is involved in tumorigenesis in various cancer types, such as bladder urothelial carcinoma (18) and colon cancer (19). TACC2, an oncogene in breast cancer, is a member of the transforming acidic coiled-coil protein family (20). BSN is mainly expressed in the brain and is involved in organizing the presynaptic cytoskeleton. BSN mutations are associated with endometrial cancer prognosis (21). TLN2 is an intracellular cytoskeletal protein, a major component of the focal adhesion complex, involved in breast cancer and hepatocellular carcinoma (22). ZDHHC8 is a member of the zinc finger DHHC domain-containing protein family that functions as a palmitoyltransferase. The role of ZDHHC8 in human cancers remains unknown. The interaction between PART1-hsa-mir-25-SLC12A5/TACC2/BSN/TLN2/ZDHHC8 is largely unclear in glioma and requires further analysis.

Our gene co-expression network analysis showed the interactions between gene molecules. We filtered the core genes and important hubs in the network module. The HLA and MT families are pivotal in the network. The HLA family plays a key role in the mechanism of anti-tumor immune effects. Cellular immunity plays a leading role in tumor cell killing, which is facilitated by humoral immunity. The HLA family includes HLA class I (HLA-A, HLA-B, HLA-C, and HLA-E) and HLA class II (HLA-DRA, HLA-DRB1, and HLA-DG). HLA-E is overexpressed in GBM (23), and HLA-DR is upregulated in gliomas compared with normal brains and is associated with poor prognosis (24). However, further in-depth studies are required to reveal the underlying mechanisms. MT family expression alterations, such as those caused by mutations in MT-ND4 or MT-ND6, involving molecular interactions and pathways that influence apoptosis are related to GBM (25). Based on GBM scRNA-seq data, Garofano et al. classified cells into four distinct clusters, including MTC (mitochondrial cluster). They specifically clarified the role of mitochondrial in GBM. General mitochondrial functions, mitochondrial metabolism, oxidative phosphorylation and fatty acid oxidation were the hallmarks of the MTC cluster. Mitochondrial complex I were upregulated in MTC cluster which related to the Warburg effect. GBM patients benefit a significantly longer survival time in the MTC group which was the only independent prognostic factor. As for tumorous immune microenvironment, microglia-like cells were enriched in MTC. MiR-30 family activated in MTC GBM patients which inhibit glycolysis and promote mitochondrial respiration. Meanwhile, MTC were sensitivity to radiotherapy and higher intracellular ROS *via* inhibiting oxidative phosphorylation (26). All these results demonstrated that mitochondrion plays a vital role in GBM, whereas, the specific role of mitochondrion also should be further examined.

Chea3 was used to predict the TFs of DEGs, and KCNIP3, PIN1, and NACC2 were found to play crucial roles in regulating DEG expression. The TF overlapping genes are presented in **Supplementary Table 6**. Zhang et al. reported that Pin1 promotes SUMO1 protein sumoylation to improve glioma malignancy (27) and clarified the role of Pin1 in glioma. Furthermore, KCNIP3 (28) and NACC2 (29) show a close relationship with cancer but whether they play related roles in glioma has not been previously reported.

GO and KEGG enrichment analyses of the biological functions enriched among the DEGs revealed GO terms involved in biological processes, including RNA catabolic processes, translational initiation, mitochondrial inner membranes, and neuron-to-neuron synapses. Some of the results of gene co-expression analysis were confirmed by the GO terms, such as mitochondrial gene and mitochondrial-related function. The results of KEGG analysis were all related to central nervous system diseases. GO and KEGG enrichment results also demonstrated the accuracy of our data analysis results.

Recently, tumor immunity has received increased attention. Tumor immunology is used to study the relationship among tumor antigens; immune function; and tumor occurrence, development, and prognosis, as well as the mechanism of the tumor immune response and tumor cell immune escape and immune-based tumor diagnosis and prevention. Our data showed that the HLA family plays a pivotal role in glioma, suggesting that tumor immunology is related to the occurrence and development of gliomas. ssGSEA to evaluate glioma microenvironment immune infiltration showed that many immune cells and immune functions exhibited aberrant infiltration and activation, respectively, in glioma. B cells and CD8+T cells showed higher and lower glioma infiltration, respectively. Han et al. observed abnormal proliferation of B cells in glioma, whereas CD8+T cell activity was suppressed (30). CD8+T cells with *Pdia3*, *Mgat5*, *Emp1*, or *Lag3* editing enhanced the survival of GBM-bearing mice (31). Lee et al. reported that local delivery of B cell-depleting anti-CD20 immunotherapy improves the OS of GBM mouse models, and that PD-L1 promotes tumoral B cell function to suppress CD8+T cell activation (32). This evidence suggests a potential role for B and CD8+T cells in glioma progression and indicates that macrophages show higher infiltration in glioma. Macrophages are recruited to the glioma environment to create a supportive stroma for glioma cell proliferation, survival, and migration through immune functions, such as the release of transforming growth factor-β, epidermal growth factor, interleukin (IL)-6, and IL-1β (33). Our results showed that neutrophils infiltrated glioma cells. IL-8, chemokine ligand 8, and macrophage migration inhibitory factor in the GBM microenvironment led to neutrophil infiltration. Neutrophils promote glioma malignancy and regulate S1004A expression to mediate glioma angiogenesis. In contrast, neutrophil depletion prolonged GBM mouse survival, suggesting that neutrophils play a significant role in glioma progression. However, the mechanism underlying the role of neutrophils in gliomas requires further study (34). Tregs are central agents responsible for modulating immunity by inhibiting CD4+T helper, CD8+T, and B cells in cancer (35). We confirmed that Treg scores were increased in glioma tissue, whereas El Andaloussi et al. reported that circulating Treg scores are increased in patients with GMB (36). The role of Tregs in glioma is currently under exploration. Stimulated cytolytic activity was observed in glioma, as demonstrated by the results of Wang et al. (37). Study showed the safe and feasible of CAR-T therapy in GBM, but CAR-T therapy also showed uncertain curative effect among GBM patients (38). Considering the limitations of CAR-T therapy, Gatto et al. firstly proposed the idea that the possibility of novel strategy CAR-M therapy in GBM (39). At present, there have been no research on the use of CAR-M in GBM patients, CAR-M therapy treat GBM need further research. Immune cytolytic activity is highly associated with glioma malignancy. The cytolytic activity-related PDGFA and EGFR genes have been found to be overexpressed in glioma, and a positive association between cytolytic activity and HLA expression has been observed; however, the underlying mechanisms are still being evaluated (37, 40). HLA also showed higher expression in glioma. The type II IFN response is a significant immune pathway found to be inhibited in the present study. IFN‐γ, the main molecule involved in the type II IFN response, plays a dual role in the immune system. IFN‐γ can inhibit tumor angiogenesis (41) and induce PD‐L1 expression to promote immune escape (42). The exact role of the type II IFN response is currently unclear. Antigen-presenting cell co-stimulation and co-inhibition, CCR, inflammation-promotion, parainflammation, T cell co-inhibition, and co-stimulation are significant immune pathways; however, their specific roles in glioma have not been investigated. ssGSEA showed that ICs expressed on immune cells were upregulated in gliomas. ICs negatively regulate T-cell activation involved in anti-tumor immune responses. Our data confirmed that the expression of ICs (B7-H3, B7-H4, B7-H5, IDO, LAG-3, PD-1, PD-L1, PD-L2, and TIM-3) was significantly higher expressed in gliomas than in normal brain tissues. Many PD-1-, PD-L1-, and IDO-targeting drugs have shown some efficacy in clinical trials, and other related ICs show potential as therapeutic targets. Researchers summarized that PD-L1 and PD-1 overexpression were associated to poorer prognosis, however, other article showed no statistical significance between PD-L1 expression and GBM prognosis. Meanwhile, the results of clinical trials demonstrated that only 7.8% of nivolumab (PD-1 inhibitor)-treated recurrent GBM patients experienced objective response compared with 23.1% in bevacizumab group, and there is no statistical significance of *de novo* MGMT-unmethylated GBM between nivolumab and standard radiotherapy compared with temozolomide and radiation therapy group. But, nivolumab could bring survival benefit for MGMT-methylated GBM patients (43). Therefore, the role of PD-L1, PD-1 and their relationship with MGMT, IDH, VISTA, B7-H3 in glioma need specific deep more research.

## Conclusions

Using a series of bioinformatic analyses, we identified DEGs and constructed a ceRNA network of prognosis-related miRNAs. We screened out the core regulatory axis to understand the occurrence and development of glioma from the perspective of the ceRNA hypothesis. The transcriptional level of regulatory axis genes was verified at the cell level, establishing a foundation for further research.

From the perspective of coding genes, co-expression network analysis of glioma DEG profiles revealed interactions between genes, reflected glioma gene expression regulation relationships, and screened out the core genes. GO and KEGG enrichment analyses were performed to determine the biological functions of different genes. To understand tumorigenesis in terms of TF-regulated coding genes, TF prediction was conducted.

Co-expression network analysis suggests that tumor immune-related genes are associated with gliomas. Further ssGSEA analysis and the expression of IC genes suggested that tumor immune disorders are closely related to glioma, providing new targets for tumor immunotherapy. Further investigation of the mechanisms of glioma pathogenesis and progression is required to yield novel glioma treatment strategies.

## Data Availability Statement

The original contributions presented in the study are included in the article/**Supplementary Material**. Further inquiries can be directed to the corresponding author.

## Ethics Statement

The studies involving human participants were reviewed and approved by Ethics Committee of Jiangyin people’s Hospital. Written informed consent for participation was not required for this study in accordance with the national legislation and the institutional requirements.

## Author Contributions

ZY and WG designed and performed the study and wrote and revised the manuscript. TZ performed cell biological experiments and analyzed related data. HG conducted the total study process. All authors contributed to the article and approved the submitted version.

## Funding

This work was supported by a grant from the Wuxi Commission of Health (Q201938) and Wuxi overseas students science and technology activity project (2019-06JY).

## Conflict of Interest

The authors declare that the research was conducted in the absence of any commercial or financial relationships that could be construed as a potential conflict of interest.

## Publisher’s Note

All claims expressed in this article are solely those of the authors and do not necessarily represent those of their affiliated organizations, or those of the publisher, the editors and the reviewers. Any product that may be evaluated in this article, or claim that may be made by its manufacturer, is not guaranteed or endorsed by the publisher.

## Abbreviations

lncRNA, long noncoding RNA; miRNA, microRNA; TCGA, The Cancer Genome Atlas; GTEx, Genotype-Tissue Expression; ceRNA, competitive endogenous RNA; GO, Gene Ontology; KEGG, Kyoto Encyclopedia of Genes and Genomes; ssGSEA, single-sample Gene Set Enrichment Analysis; IC, immune checkpoint; GMB, glioblastoma; DEGs, differentially expressed genes; TF, transcription factor; TMSB10, thymosin beta 10; DGIDB, Drug Gene Interaction Database; SLC12A5, solute carrier family 12 member 5; TACC2, transforming acidic coiled-coil 2; BSN, bassoon presynaptic cytomatrix protein; TLN2, Talin 2; ZDHHC8, zinc finger DHHC-type palmitoyltransferase 8; OS, overall survival; KCNIP3, potassium voltage-gated channel interacting protein 3; MT, mitochondrial transcription.

## References

[1] Ostrom QT Patil N Cioffi G Waite K Kruchko C Barnholtz-Sloan JS. CBTRUS Statistical Report: Primary Brain and Other Central Nervous System Tumors Diagnosed in the United States in 2013-2017. Neuro Oncol (2020) 22(12 Suppl 2):iv1–iv96. doi: 10.1093/neuonc/noaa200 33123732 PMC7596247

[2] Weller M van den Bent M Preusser M Le Rhun E Tonn JC Minniti G. EANO Guidelines on the Diagnosis and Treatment of Diffuse Gliomas of Adulthood. Nat Rev Clin Oncol (2021) 18(3):170–86. doi: 10.1038/s41571-020-00447-z PMC7904519 33293629

[3] Franceschi E Tosoni A Bartolini S Minichillo S Mura A Asioli S. Histopathological Grading Affects Survival in Patients With IDH-Mutant Grade II and Grade III Diffuse Gliomas. Eur J Cancer (2020) 137:10–7. doi: 10.1016/j.ejca.2020.06.018 32721633

[4] Gao Y Weenink B van den Bent MJ Erdem-Eraslan L Kros JM Sillevis Smitt P. Expression-Based Intrinsic Glioma Subtypes are Prognostic in Low-Grade Gliomas of the EORTC22033-26033 Clinical Trial. Eur J Cancer (2018) 94:168–78. doi: 10.1016/j.ejca.2018.02.023 29571083

[5] Stylli SS. Novel Treatment Strategies for Glioblastoma. Cancers (Basel) (2020) 12(10):2883–94. doi: 10.3390/cancers12102883 PMC7599818 33049911

[6] Balihodzic A Barth DA Prinz F Pichler M. Involvement of Long Non-Coding RNAs in Glucose Metabolism in Cancer. Cancers (Basel) (2021) 13(5):977–98. doi: 10.3390/cancers13050977 PMC7956509 33652661

[7] Chen X You ZH Yan GY Gong DW. IRWRLDA: Improved Random Walk With Restart for lncRNA-Disease Association Prediction. Oncotarget (2016) 7(36):57919–31. doi: 10.18632/oncotarget.11141 PMC5295400 27517318

[8] Momtazmanesh S Rezaei N. Long Non-Coding RNAs in Diagnosis, Treatment, Prognosis, and Progression of Glioma: A State-Of-The-Art Review. Front Oncol (2021) 11:712786. doi: 10.3389/fonc.2021.712786 34322395 PMC8311560

[9] Ebrahimpour A Sarfi M Rezatabar S Tehrani SS. Novel Insights Into the Interaction Between Long non-Coding RNAs and microRNAs in Glioma. Mol Cell Biochem (2021) 476(6):2317–35. doi: 10.1007/s11010-021-04080-x 33582947

[10] Salmena L Poliseno L Tay Y Kats L Pandolfi PP. A ceRNA Hypothesis: The Rosetta Stone of a Hidden RNA Language? Cell (2011) 146(3):353–8. doi: 10.1016/j.cell.2011.07.014 PMC3235919 21802130

[11] Ritchie ME Phipson B Wu D Hu Y Law CW Shi W. Limma Powers Differential Expression Analyses for RNA-Sequencing and Microarray Studies. Nucleic Acids Res (2015) 43(7):e47. doi: 10.1093/nar/gkv007 25605792 PMC4402510

[12] Yu G Wang LG Han Y He QY. Clusterprofiler: An R Package for Comparing Biological Themes Among Gene Clusters. OMICS (2012) 16(5):284–7. doi: 10.1089/omi.2011.0118 PMC3339379 22455463

[13] Ostrom QT Cioffi G Gittleman H Patil N Waite K Kruchko C. CBTRUS Statistical Report: Primary Brain and Other Central Nervous System Tumors Diagnosed in the United States in 2012-2016. Neuro Oncol (2019) 21(Suppl 5):v1–v100. doi: 10.1093/neuonc/noz150 31675094 PMC6823730

[14] Gatto L Franceschi E Di Nunno V Tosoni A Lodi R Brandes AA. Liquid Biopsy in Glioblastoma Management: From Current Research to Future Perspectives. Oncologist (2021) 26:1–14. doi: 10.1002/onco.13858 34105205 PMC8488799

[15] Karreth FA Pandolfi PP. ceRNA Cross-Talk in Cancer: When Ce-Bling Rivalries Go Awry. Cancer Discovery (2013) 3(10):1113–21. doi: 10.1158/2159-8290.CD-13-0202 PMC3801300 24072616

[16] Jin Z Piao L Sun G Lv C Jing Y Jin R. Long Non-Coding RNA PART1 Exerts Tumor Suppressive Functions in Glioma via Sponging miR-190a-3p and Inactivation of PTEN/AKT Pathway. Onco Targets Ther (2020) 13:1073–86. doi: 10.2147/OTT.S232848 PMC7007780 32099409

[17] Peng G Yuan X Yuan J Liu Q Dai M Shen C. miR-25 Promotes Glioblastoma Cell Proliferation and Invasion by Directly Targeting NEFL. Mol Cell Biochem (2015) 409(1-2):103–11. doi: 10.1007/s11010-015-2516-x 26209061

[18] Liu JY Dai YB Li X Cao K Xie D Tong ZT. Solute Carrier Family 12 Member 5 Promotes Tumor Invasion/Metastasis of Bladder Urothelial Carcinoma by Enhancing NF-Kappab/MMP-7 Signaling Pathway. Cell Death Dis (2017) 8(3):e2691. doi: 10.1038/cddis.2017.118 28333147 PMC5386524

[19] Yu C Yu J Yao X Wu WK Lu Y Tang S. Discovery of Biclonal Origin and a Novel Oncogene SLC12A5 in Colon Cancer by Single-Cell Sequencing. Cell Res (2014) 24(6):701–12. doi: 10.1038/cr.2014.43 PMC4042168 24699064

[20] Cheng S Douglas-Jones A Yang X Mansel RE Jiang WG. Transforming Acidic Coiled-Coil-Containing Protein 2 (TACC2) in Human Breast Cancer, Expression Pattern and Clinical/Prognostic Relevance. Cancer Genomics Proteomics (2010) 7(2):67–73. 20335520

[21] Qiao Z Jiang Y Wang L Wang L Jiang J Zhang J. Mutations in KIAA1109, CACNA1C, BSN, AKAP13, CELSR2, and HELZ2 Are Associated With the Prognosis in Endometrial Cancer. Front Genet (2019) 10:909. doi: 10.3389/fgene.2019.00909 31787999 PMC6854026

[22] Malla RR Vempati RK. Talin: A Potential Drug Target for Cancer Therapy. Curr Drug Metab (2020) 21(1):25–32. doi: 10.2174/1389200221666200214114018 32056520

[23] Kren L Slaby O Muckova K Lzicarova E Sova M Vybihal V. Expression of Immune-Modulatory Molecules HLA-G and HLA-E by Tumor Cells in Glioblastomas: An Unexpected Prognostic Significance? Neuropathology (2011) 31(2):129–34. doi: 10.1111/j.1440-1789.2010.01149.x 20667016

[24] Diao J Xia T Zhao H Liu J Li B Zhang Z. Overexpression of HLA-DR is Associated With Prognosis of Glioma Patients. Int J Clin Exp Pathol (2015) 8(5):5485–90. PMC4503125 26191254

[25] Nagy A Eder K Selak MA Kalman B. Mitochondrial Energy Metabolism and Apoptosis Regulation in Glioblastoma. Brain Res (2015) 1595:127–42. doi: 10.1016/j.brainres.2014.10.062 25451120

[26] Garofano L Migliozzi S Oh YT D'Angelo F Najac RD Ko A. Pathway-Based Classification of Glioblastoma Uncovers a Mitochondrial Subtype With Therapeutic Vulnerabilities. Nat Cancer (2021) 2(2):141–56. doi: 10.1038/s43018-020-00159-4 PMC7935068 33681822

[27] Zhang A Tao W Zhai K Fang X Huang Z Yu JS. Protein Sumoylation With SUMO1 Promoted by Pin1 in Glioma Stem Cells Augments Glioblastoma Malignancy. Neuro Oncol (2020) 22(12):1809–21. doi: 10.1093/neuonc/noaa150 PMC7746948 32592588

[28] Zhou X Xiao C Han T Qiu S Wang M Chu J. Prognostic Biomarkers Related to Breast Cancer Recurrence Identified Based on Logit Model Analysis. World J Surg Oncol (2020) 18(1):254. doi: 10.1186/s12957-020-02026-z 32977823 PMC7519567

[29] Shivakumar M Lee Y Bang L Garg T Sohn KA Kim D. Identification of Epigenetic Interactions Between miRNA and DNA Methylation Associated With Gene Expression as Potential Prognostic Markers in Bladder Cancer. BMC Med Genomics (2017) 10(Suppl 1):30. doi: 10.1186/s12920-017-0269-y 28589857 PMC5461531

[30] Han S Feng S Ren M Ma E Wang X Xu L. Glioma Cell-Derived Placental Growth Factor Induces Regulatory B Cells. Int J Biochem Cell Biol (2014) 57:63–8. doi: 10.1016/j.biocel.2014.10.005 25450457

[31] Ye L Park JJ Dong MB Yang Q Chow RD Peng L. In Vivo CRISPR Screening in CD8 T Cells With AAV-Sleeping Beauty Hybrid Vectors Identifies Membrane Targets for Improving Immunotherapy for Glioblastoma. Nat Biotechnol (2019) 37(11):1302–13. doi: 10.1038/s41587-019-0246-4 PMC6834896 31548728

[32] Lee-Chang C Rashidi A Miska J Zhang P Pituch KC Hou D. Myeloid-Derived Suppressive Cells Promote B Cell-Mediated Immunosuppression via Transfer of PD-L1 in Glioblastoma. Cancer Immunol Res (2019) 7(12):1928–43. doi: 10.1158/2326-6066.CIR-19-0240 PMC6891201 31530559

[33] Hambardzumyan D Gutmann DH Kettenmann H. The Role of Microglia and Macrophages in Glioma Maintenance and Progression. Nat Neurosci (2016) 19(1):20–7. doi: 10.1038/nn.4185 PMC4876023 26713745

[34] Khan S Mittal S McGee K Alfaro-Munoz KD Majd N Balasubramaniyan V. Role of Neutrophils and Myeloid-Derived Suppressor Cells in Glioma Progression and Treatment Resistance. Int J Mol Sci (2020) 21(6):1954–72. doi: 10.3390/ijms21061954 PMC7139844 32182988

[35] See AP Parker JJ Waziri A. The Role of Regulatory T Cells and Microglia in Glioblastoma-Associated Immunosuppression. J Neurooncol (2015) 123(3):405–12. doi: 10.1007/s11060-015-1849-3 26123363

[36] El Andaloussi A Lesniak MS. An Increase in CD4+CD25+FOXP3+ Regulatory T Cells in Tumor-Infiltrating Lymphocytes of Human Glioblastoma Multiforme. Neuro Oncol (2006) 8(3):234–43. doi: 10.1215/15228517-2006-006 PMC1871953 16723631

[37] Wang ZL Wang Z Li GZ Wang QW Bao ZS Zhang CB. Immune Cytolytic Activity Is Associated With Genetic and Clinical Properties of Glioma. Front Immunol (2019) 10:1756. doi: 10.3389/fimmu.2019.01756 31428092 PMC6688525

[38] O'Rourke DM Nasrallah MP Desai A Melenhorst JJ Mansfield K Morrissette JJD. A Single Dose of Peripherally Infused EGFRvIII-Directed CAR T Cells Mediates Antigen Loss and Induces Adaptive Resistance in Patients With Recurrent Glioblastoma. Sci Transl Med (2017) 9(399). doi: 10.1126/scitranslmed.aaa0984 PMC5762203 28724573

[39] Gatto L Nunno VD Franceschi E Brandes AA. Chimeric Antigen Receptor Macrophage for Glioblastoma Immunotherapy: The Way Forward. Immunotherapy (2021) 13(11):879–83. doi: 10.2217/imt-2021-0054 34078139

[40] Rooney MS Shukla SA Wu CJ Getz G Hacohen N. Molecular and Genetic Properties of Tumors Associated With Local Immune Cytolytic Activity. Cell (2015) 160(1-2):48–61. doi: 10.1016/j.cell.2014.12.033 25594174 PMC4856474

[41] Mitra-Kaushik S Harding J Hess J Schreiber R Ratner L. Enhanced Tumorigenesis in HTLV-1 Tax-Transgenic Mice Deficient in Interferon-Gamma. Blood (2004) 104(10):3305–11. doi: 10.1182/blood-2004-01-0266 15292059

[42] Qian J Wang C Wang B Yang J Wang Y Luo F. The IFN-Gamma/PD-L1 Axis Between T Cells and Tumor Microenvironment: Hints for Glioma Anti-PD-1/PD-L1 Therapy. J Neuroinflamm (2018) 15(1):290. doi: 10.1186/s12974-018-1330-2 PMC6192101 30333036

[43] Di Nunno V Franceschi E Gatto L Bartolini S Brandes AA. Predictive Markers of Immune Response in Glioblastoma: Hopes and Facts. Future Oncol (2020) 16(15):1053–63. doi: 10.2217/fon-2020-0047 32270715

